# The accessory heads of the quadriceps femoris muscle may affect the layering of the quadriceps tendon and potential graft harvest lengths

**DOI:** 10.1007/s00167-023-07647-x

**Published:** 2023-11-06

**Authors:** Łukasz Olewnik, Nicol Zielinska, Paloma Aragones, Kacper Ruzik, Friedrich Paulsen, Andrzej Borowski, Robert F. LaPrade

**Affiliations:** 1 Department of Anatomical Dissection and Donation, Medical University of Lodz, Lodz, Poland; 2 Institute of Functional and Clinical Anatomy, Friedrich Alexander University Erlangen-Nürnberg, Erlangen, Germany; 3 Orthopaedics and Pediatric Orthopaedics Department, Medical University of Lodz, 90-419 Lodz, Poland; 4 Twin Cities Orthopedics, Edina, MN USA; 5 Department of Anatomy and Embryology, School of Medicine, Complutense University of Madrid, Madrid, Spain

**Keywords:** Quadriceps tendon, Harvest, Accessory heads, Quadriceps femoris, Surgery, Sport

## Abstract

**Purpose:**

The aim of the study was to assess the quadriceps femoris system for the presence of additional layers.

**Methods:**

One hundred and twenty-eight lower limbs fixed in 10% formalin were examined.

**Results:**

Five types of quadriceps tendon layering were found based on the accessory heads of the quadriceps muscle. Type I (55%)—represented by four heads and four layers, and it was something new because standard orthopaedic textbooks described quadriceps tendon as a structure composed of only three layers. Type II (27.4%)—the first four layers were the same as in Type 1, but the accessory tendon of the fifth head of the quadriceps femoris muscle had the deepest attachments. Type III (10.9%)—this type included 6 heads of quadriceps femoris. It consisted of five layers. Type IV (3.1%)—this type included 7 quadriceps femoris heads. This type consisted of only four layers. Type V (3.1%)—this type included 8 heads of the quadriceps femoris heads. This type consist of 5 layers.

**Conclusion:**

The findings of this study provide a detailed anatomy of the quadriceps tendon including the accessory tendons of the accessory heads of the quadriceps tendon. The accessory heads of the quadriceps femoris muscle contribute to the layering of the quadriceps tendon. The second conclusion of this study is the development of safe distances depending on the types. Not all types are perfect for harvesting—Type IV seems to be the safest type, in turn Type V the most dangerous.

**Supplementary information:**

**Supplementary information** accompanies this paper at 10.1007/s00167-023-07647-x.

## Introduction

Although, it might seem that such a large muscle as the quadriceps femoris (QF) has already been completely studied, it is not true. In fact, its proximal and distal attachments demonstrate considerable variability, and the relations of its parts to each other are different [6–8, 16, 28].

Grob et al. [8] were the first to describe the fifth head of the QF and assign it the name *tensor vastus intermedius*. Its prevalence was estimated at 100% [7]. Later studies on the QF carried out by Olewnik et al. [17] showed that this muscle can have five, six, seven, or even eight heads, which completely gave a new morphologically picture of this muscle. They estimated the presence of additional heads at 64.1%.

A study of the architectural structure of the QF found that the vastus lateralis consists of three layers: superficial, intermediate, and deep [16]. Interestingly, only the superficial and intermediate layers are part of the quadriceps tendon (QT), while the deep layer attaches laterally to the patella. The vastus medialis consists of two parts: longus and obliquus [16].

While it might seem that the quadriceps femoris tendon has been thoroughly understood, nothing could be further from the truth. Most orthopaedic textbooks or scientific studies indicate that the QF tendon consists of superficial, intermediate, and deep layers [1, 8, 9, 26]. Recent studies by Olewnik et al. [18] indicate that the QF tendon consists of four layers, not three, which may be significantly important for surgical procedures in the knee joint (for example, reconstructing anterior cruciate ligament (ACL). No consensus exists on the tendons or ligaments to use for reconstructing anterior cruciate ligament rupture; however, graft harvest from the QT has some advantages. The most commonly used grafts for ACL reconstruction are bone–patellar tendon–bone and hamstring tendon autografts [18, 26]. Despite this, in the case of hamstring grafts, additional bands are often present in the gracilis and semitendinosus tendons, and this may lead to tendon fibrosis. However, patients receiving bone–patellar tendon–bone grafts are more likely to experience donor site morbidity, including kneeling pain, graft harvest site pain, and sensory loss [4, 5, 13, 14, 21, 26, 30]. There is, hence, great interest in using QT grafts for ACL reconstruction, as this is associated with less pain at the harvest site, and a lower risk of graft failure than for hamstring grafts [19, 26].

The current literature provided information that the safe harvest distance of the QT is 5.5 to 8 cm in length [21, 23, 24, 26]. This was confirmed in a recent study by Olewnik et al. [18], namely harvesting QTs in men up to 105 mm seems safe, and in women up to 80 mm.

The purpose of this study was to qualitatively and quantitatively describe the anatomy of the QT including its size, accessory tendons of the accessory heads of the QF, layers and the relationship between layers.

## Materials and methods

One hundred and twenty-eight lower limbs (64 woman and 64 men) fixed in 10% formalin were examined. The mean age “at death” of the cadavers was 81.8 years in men, and 80.5 years in women. The cadavers were the property of the Department of Anatomical Dissection and Donation of the Medical University of Lodz, Poland, following donation to the university anatomy programme, and of the Donors and Dissecting Rooms Center, Universidad Complutense de Madrid, Spain. Informed consent was obtained from all donors before they died. The Bioethics Committee of the Medical University of Lodz (resolution RNN/114/19/KE) approved the study protocol. Lower limbs with evidence of surgical intervention in the dissected area were excluded. The thigh area was dissected as described previously [14–16, 18].

The lower limb was positioned on the dissection table in the supine position. First, the skin of the thigh was cut along with the subcutaneous tissue, and the inguinal ligament was identified. Following this, all femoral nerve branches were dissected. The sartorius and rectus femoris muscles were transacted in the middle of the muscle belly and lifted to optimise the deep vasti view. The rectus femoris was cleaned from the proximal attachments to the distal attachment. Each of the vasti bellies was dissected to reveal each muscle belly’s origin and insertion. The next step was to see if there were any additional QF heads. If there were any, the muscle bellies were carefully cleaned in the direction of the transition to the tendon. Finally, the tendons were dissected to identify potential additional tendon bands. The tendons were thoroughly cleaned and checked for layering. The places of attachment and layering of the tendons of the additional QF heads were thoroughly assessed. Then, the muscle was excised along with the tendons and its layering was assessed from the inside.

Upon dissection, the following morphological features of the QF tendon were assessed:

- The presence of accessory heads of the QF
- The arrangement of the layers of each of the tendons
- Morphometric measurements of the tendons of the QF (length, insertion width, and thickness)
- Assessment of the potential safe length, width and thickness of a QT harvest.

An electronic digital calliper was used for all measurements (Mitutoyo Corporation, Kawasaki-shi, Kanagawa, Japan), and each measurement was performed twice with an accuracy of up to 0.01 mm.

### Statistical analysis

Statistical analysis was performed for the anatomical part. Descriptive statistics were used to characterise the morphology of the QF. The Shapiro–Wilk test assessed normality of continuous data distribution. It was nonparametric data; hence, differences in morphological parameters between sexes and body sides were compared with the Manny–Whitney test and the Wilcoxon sing rank test, respectively. The Kruskal–Willis test was used to evaluate the individual parameters between the different types.

## Results

Five types of QT layering were found depending on the accessory heads of the quadriceps muscle.

**Fig. 1:**
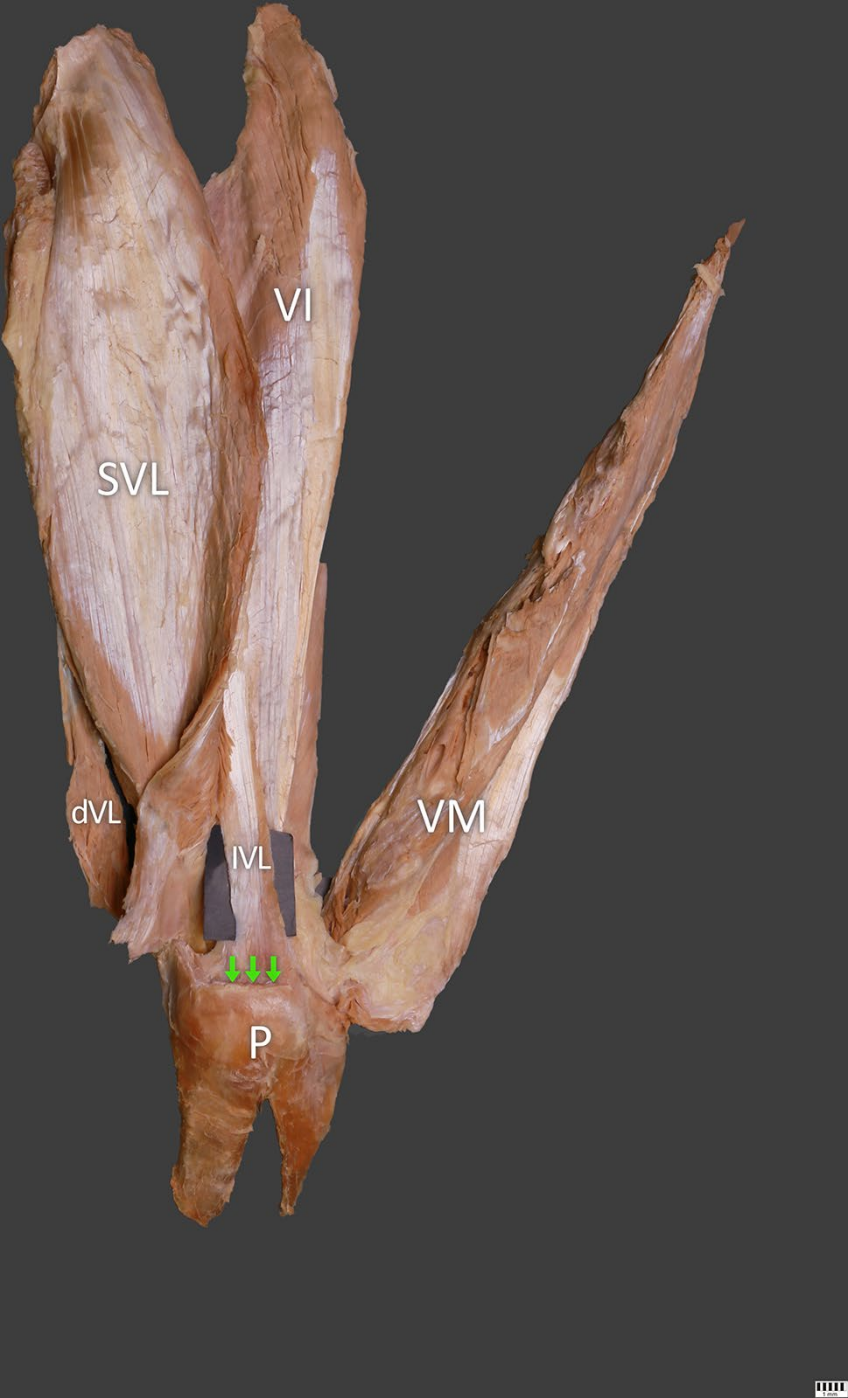
Type I of the quadriceps femoris tendon. Anterior view. Right side. *SVL* superficial part of the vastus lateralis, *IVL* intermediate part of the vastus lateralis, *dVL* deep part of the vastus lateralis, *VM* vastus medialis, *P* patella

Type I (55.5%)—This type was found in 71 cases. It consisted of four layers: the first layer (superficial) was formed by the rectus femoris tendon and fascia. The second (middle) was composed of the vastus medialis and superficial part of the vastus lateralis muscle. The third layer (middle deep) was composed of the intermediate part of the vastus lateralis muscle. Finally, the fourth layer (the deep layer) was composed of the vastus intermedius. The mean length of the QT was 109.55 mm—Fig. 1.

**Fig. 2:**
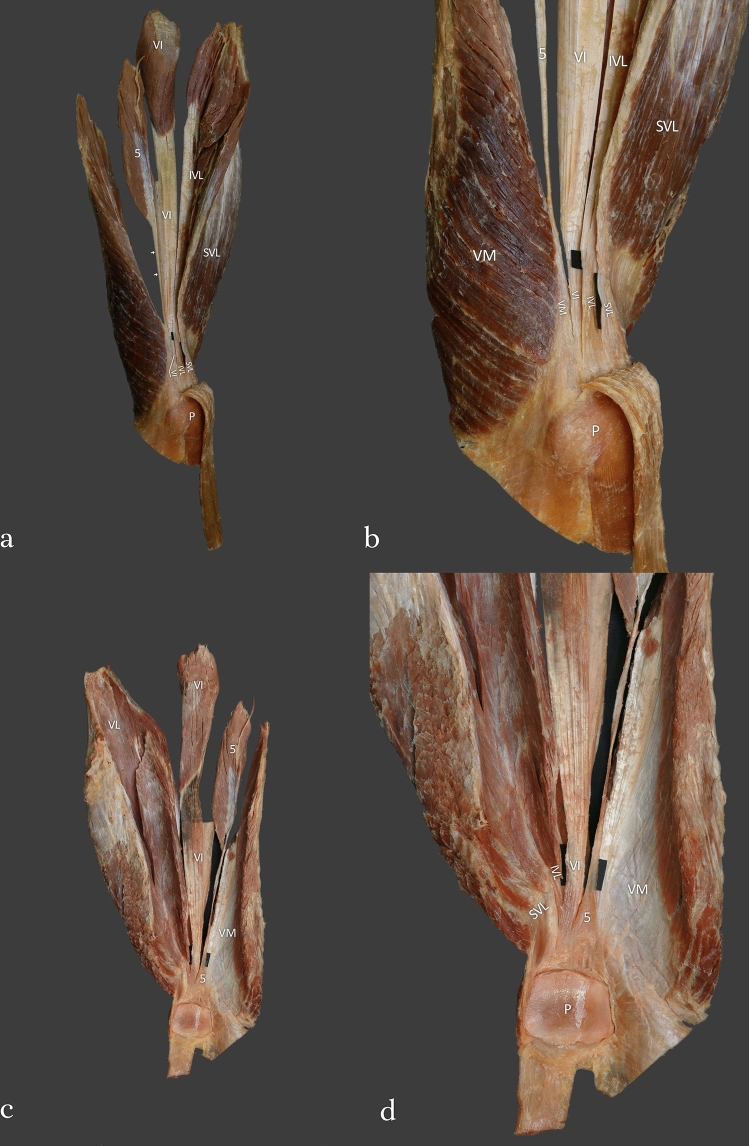
**a** Type II of the quadriceps femoris tendon. Anterior view of the quadriceps femoris. Left side. *SVL* superficial part of the vastus lateralis, *IVL* intermediate part of the vastus lateralis, *VI* vastus intermedius, *5* fifth head of the quadriceps femoris, *P* patella. **b** Type II of the quadriceps femoris tendon. Enlarged photo. Anterior view of the quadriceps femoris. Left side. *SVL* superficial part of the vastus lateralis, *IVL* intermediate part of the vastus lateralis, *VI* vastus intermedius, *5* fifth head of the quadriceps femoris, *P* patella. **c** Type II of the quadriceps femoris tendon. Posterior view of the quadriceps femoris. Left side, *VI* vastus intermedius, *5* fifth head of the quadriceps femoris, *P* patella. *VM* vastus medialis. **d** Type II of the quadriceps femoris tendon. Enlarged photo. Posterior view of the quadriceps femoris. Left side, *VI* vastus intermedius, *5* fifth head of the quadriceps femoris, *P* patella. *VM* vastus medialis. *SVL* superficial part of the vastus lateralis, *IVL* intermediate part of the vastus lateralis

Type II (27.4%)—This type was found in 35 cases. The first four layers were the same as type 1, but the accessory tendon of the fifth head of the QF had the deepest attachments. The mean QT length was 88.81 mm—Fig. 2a–d.

**Fig. 3:**
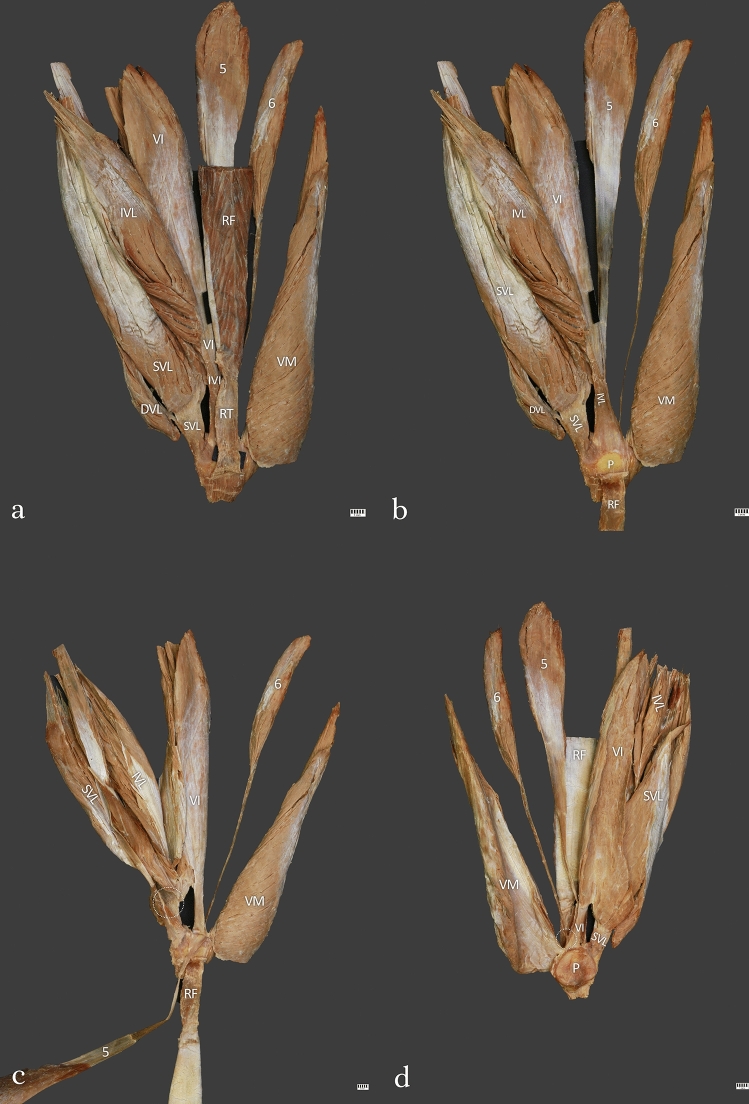
**a** Type III of the quadriceps femoris tendon. Anterior view. Right side. *RF* rectus femoris, *RT* rectus tendon, *SVL* superficial part of the vastus lateralis, *IVL* intermediate part of the vastus lateralis, *DVL* deep part of the vastus lateralis, *VI* vastus intermedius, *5* fifth head of the quadriceps femoris, *6* sixth head of the quadriceps femoris. **b** Type III of the quadriceps femoris tendon. Isolated rectus femoris. Anterior view. Right side. *RF* rectus femoris, *SVL* superficial part of the vastus lateralis, *IVL* intermediate part of the vastus lateralis, *DVL* deep part of the vastus lateralis, *VI* vastus intermedius, *5* fifth head of the quadriceps femoris, *6 *sixth head of the quadriceps femoris. **c** Type III of the quadriceps tendon. Isolated rectus femoris and fifth head of the quadriceps femoris. *RF* rectus femoris, *SVL* superficial part of the vastus lateralis, *IVL* intermediate part of the vastus lateralis, *VI* vastus intermedius, *VM* vastus medialis, *5* fifth head of the quadriceps femoris, *6* sixth head of the quadriceps femoris. **d** Type III of the quadriceps femoris tendon. Posterior view. Right side. *RF* rectus femoris, *SVL* superficial part of the vastus lateralis, *IVL* intermediate part of the vastus lateralis, *DVL* deep part of the vastus lateralis, *VI* vastus intermedius, *5* fifth head of the quadriceps femoris, *6* sixth head of the quadriceps femoris

Type III (10.9%)—This type was found in 14 cases. This type included six QF heads. It consisted of five layers. The first layer was composed of the rectus femoris and fascia. The second layer was formed by the superficial and intermediate part of the vastus lateralis and vastus medialis. The third layer consisted of the tendon of the fifth head of the QF. The fourth layer was formed by vastus intermedius, and the innermost part (the fifth layer) was formed by the tendon of the sixth head of the QF. The mean length of the QT was 78.77 mm—Fig. 3–d.

**Fig. 4:**
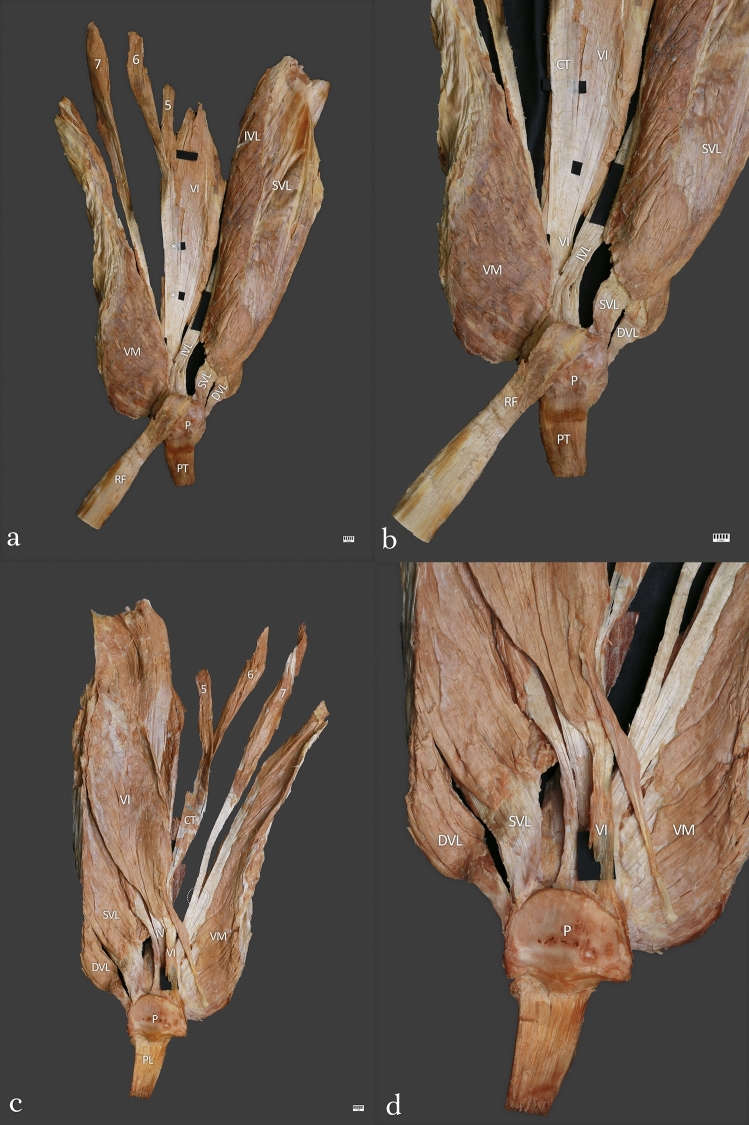
**a** Type IV of the quadriceps femoris tendon. Anterior view. Left side. *RF* rectus femoris, *P* patella, *PT* patella tendon, *VM* vastus medialis, *SVL* superficial part of the vastus lateralis, *IVL* intermediate part of the vastus lateralis, *DVL* deep part of the vastus lateralis, *VI* vastus intermedius, *5* fifth head of the quadriceps femoris, *6* sixth head of the quadriceps femoris, *7* seventh head of the quadriceps femoris *common tendon for the fifth and sixth head of the quadriceps femoris. **b** Type IV of the quadriceps femoris tendon. Enlarged photo. Anterior view. Left side. *P* patella, *PT* patella tendon, *RF* – rectus femoris, *VI* vastus intermedius, *CT* common tendon, *IVL* intermediate part of the vastus lateralis, *SVL* superficial part of the vastus lateralis, *DVL* deep part of the vastus lateralis. **c** Type IV of the quadriceps femoris tendon. Posterior view. Left side. *P* patella, *PT* patella tendon, *VI* -vastus intermedius, *SVL* superficial part of the vastus lateralis, *IVL* intermediate part of the vastus lateralis, *VM* vastus medialis, *CT* common tendon, *5* fifth head of the quadriceps femoris, *6* sixth head of the quadriceps femoris, *7* seventh head of the quadriceps femoris. **d** Type IV of the quadriceps femoris tendon. Enlarged photo. Posterior view. Left side. *DVL* deep part of the vastus lateralis, *SVL* superficial part of the vastus lateralis, *VI* vastus intermedius. *VM* vastus medialis

Type IV (3.1%)—This type was found in 4 cases. This type included seven QF heads. This type consisted of only four layers. The first layer was formed by the rectus femoris, the second layer was formed by the superficial part of the vastus lateralis and vastus medialis, and the tendon of the seventh head of the QF. The third layer was formed by the intermediate part of the vastus lateralis. The fourth layer is a fusion between the accessory tendons of the fifth and sixth heads of the QF with the vastus intermedius. The mean length of the QT was 134.46 mm—Fig. 4a–d.

**Fig. 5:**
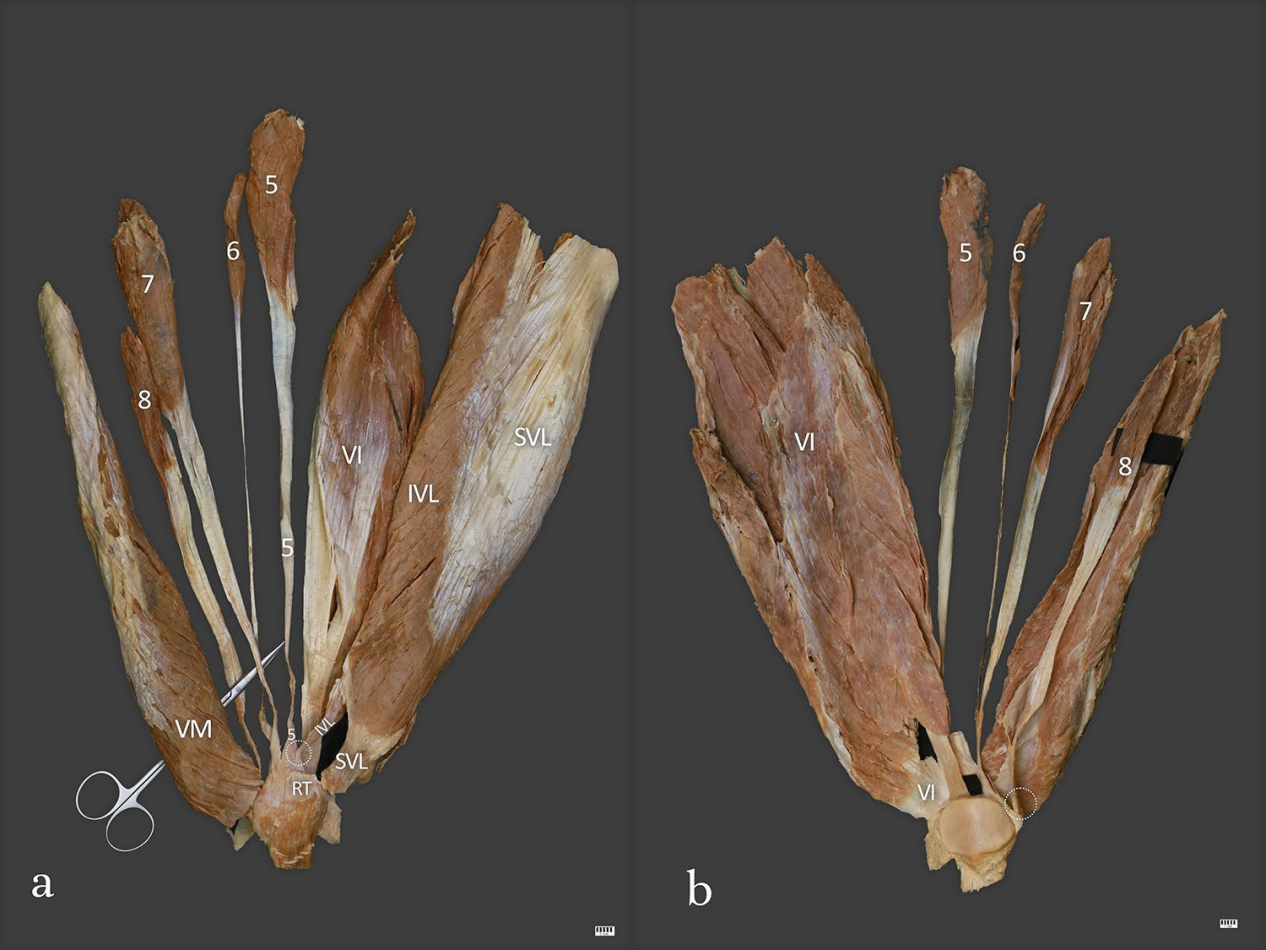
**a** Type V of the quadriceps femoris tendon. Anterior view. Right side. *SVL* superficial part of the vastus lateralis, *IVL* intermediate part of the vastus lateralis, *VI* vastus intermedius, *RT* rectus tendon, *VM* vastus medialis, *5* fifth head of the quadriceps femoris, *6* sixth head of the quadriceps femoris, *7* seventh head of the quadriceps femoris, *8* eight head of the quadriceps femoris, white circle shows the connection between fifth head tendon and intermediate part of the vastus lateralis. **b** Type V of the quadriceps femoris tendon. Posterior view. Right side. *VI* vastus intermedius, *5* fifth head of the quadriceps femoris, *6* sixth head of the quadriceps femoris, *7* seventh head of the quadriceps femoris, *8* eight head of the quadriceps femoris, white circle shows the connection between fifth head tendon and intermediate part of the vastus lateralis

Type V (3.1%)—This type was found in 4 cases. It included eight heads of the QF heads. This type consisted of five layers. The first layer was composed by the rectus femoris. The second layer was composed of the superficial part of the vastus lateralis and vastus medialis. The third layer was composed by the intermediate part of the vastus lateralis and tendon of the fifth and sixth heads of the QF. The fourth layer was composed of the tendon of the seventh head of the QF, while the fifth layer was composed of the vastus intermedius. The tendon of the eight head of the QF was attached to the medial part of the patella. The mean length of the QT was 27.92 mm—Fig. 5a, b.

Morphometric measurements between sexes are presented in Supplementary Table 1, and between the sides of the body in Supplementary Table 2. The relationships between the types are presented in Supplementary Table 3.

## Discussion

The most important finding of the present work was that it presents a comprehensive analysis of the quantitative anatomy of the QT layers based on anatomical dissections, including the accessory heads and tendons of the QF. It also provides the distance between the tendon connections and the distance from the patella for the various classifications. All previous publications were based on the determination of layering only, without specifying additional heads (fifth, sixth, seventh, eighth).

Much research has been performed on QT layers [1, 7, 9, 27, 31]. These are variable reports as to the number of layers, some indicating only two or three layers, and more recent studies indicating as many as four. Some believe that the number of layers may vary for each person. Waligora et al. [27] reported that the QT consisted of three layers, but layering was produced by different muscle components as their tendons can be bilaminar or trilaminar. For example, the rectus femoris can produce both a superficial layer and an intermediate layer, and the vastus medialis can produce both an intermediate layer and a deep layer.

On the other hand, Zeiss et al. [31] reported that a QT can have two to four layers, with two layers present in 30% of cases, three in 56% and four in 6%. In 8%, the laminations were barely perceptible; they also noted that if two QT layers were present, the superficial one was composed of the rectus femoris and vastus medialis and lateralis, and the deep layer was composed of the vastus intermedius. If there was a three-layer QT, then the superficial layer was composed of the rectus femoris, the intermediate layer of the vastus medialis and vastus lateralis, and the deep layer of the vastus intermedius [31]. On the other hand, in the case of a four-layer QT, then this intermediate layer formed by the vastus medialis and vastus lateralis was bilaminar [31].

In turn, Andrikoula et al. [1], Iriuchishima et al. [9], Sonin et al. [24], and Yablon et al. [29] report a three-layered QT. In all studies, the three layers were identically composed, i.e. the rectus femoris formed the superficial layer, while the vastus medialis and vastus lateralis formed the intermediate layer, and the vastus intermedius formed a deep layer; this was confirmed by Zeiss et al. [31] for all three layers.

Of particular interest, Grob et al. [8] reported that six elements constitute the QT, including the lateral aponeurosis of the vastus intermedius, superficial and deep medial aponeurosis of the vastus intermedius, vastus lateralis, tensor vastus intermedius, and rectus femoris [8]. The superficial layer was composed of the rectus femoris, while the intermediate layer was composed of the medial superficial and deep aponeurosis of the vastus intermedius and vastus lateralis and tensor vastus intermedius, and the deep layer was composed of the lateral vastus intermedius. Interestingly, the authors concluded that the vastus medialis was not part of the QT and inserted onto the aponeurosis of the vastus intermedius and tendon of the rectus femoris.

It is arguably wrong to include the tensor vastus intermedius in layers without any specified types due to the fact that it is not always present. Grob et al. [7] found it to occur in all limbs, although their research sample was small. On the other hand, Olewnik et al. [17] found that accessory heads were present in 64.1% of tested samples. Bonnechere et al. [3] also found no accessory heads. Olewnik et al. [18] indicate in the earliest studies that the QT consists of four layers, and not three as previously thought. The difference in research was that vasci muscles have a more complex structure than previously expected [16]. The vastus lateralis consists of three parts: superficial, intermediate, and deep [16]. Both the superficial and intermediate parts are part of the quadriceps tendon [18]. Previously, no one paid any attention to it. Both anatomical and MRI examinations revealed the presence of four layers [18]. The first layer (superficial) was formed by the rectus femoris tendon and fascia, the second layer of the vastus medialis and superficial part of the vastus lateralis tendons (the superficial part of the vastus lateralis and vastus medialis were blended in the distal part), the third by the intermediate part of the vastus lateralis, and the fourth layer by the tendon of the vastus intermedius.

Summarising the information, QT layering should be systematised. An effective classification should include both layers without accessory heads and with accessory heads. The present study has yielded significant results, repeatability between given types. Our Type I confirms previous studies of the tendon with only four parts of the QF. Type II consisted of five layers, although it should be noted that the tendon of the accessory head (fifth head) did not participate in the formation of the QT, but had a much deeper attachment. Type III exhibited a very interesting layering with two accessory QF heads. Most importantly, the second layer consisted of three anatomical elements, viz*.* the superficial and intermediate part of the vastus lateralis and the vastus medialis, which has not been described previously. Another important findings was that the tendon of the second accessory head (the sixth), like in Type II, was independent and attached to the deepest layer.

Type IV was an interesting type characterised by three additional heads (i.e. seven heads). The name of the fifth and sixth heads was joined together and was fused with the vastus intermedius, while the seventh head was fused with the vastus medialis. This type ideally predisposes to the possibility of tendon harvest for, e.g. ACL recruitment, due to the fact that no tendon attaches separately and the tendons blended together to form an uniform QT. Type V, a very rare type, was characterised by an accessory four heads (i.e. eight heads). The tendon of the fifth and sixth heads of the QF together with the intermediate part of the vastus lateralis formed the third layer of the QT, while the tendon of the seventh head formed the independent fourth layer of the QT. The tendon of the eighth head had an interesting site for its distal attachment—the medial side of the patella.

It is also very important to know the appropriate possible length of the QT to optimise a QT graft harvest. The current literature reports that the safe harvest of QT ranges from 5.5 to 8 cm. [4, 5, 12, 13, 20–23, 25, 30]. Olewnik et al. [18] suggested that harvesting tendons appears safe up to 105 mm in men, and up to 80 mm in women. Therefore, surgeons should be careful when they operated on women because the length is much shorter. Current research shows that a global and comprehensive approach to the complexity of a structure such as the entire QF is important. It is not only possible to focus on its layers and the fact that it usually consists of four heads, but it is necessary to take into account the presence of accessory tendons of accessory heads because they occur quite often. It is estimated that in Type I, which consists of four layers, the average distance between the tendons and the patella is 109.55 mm. This type of layering and the absence of accessory tendons, as well as the long distance between the tendons and the patella, appear to be safe for QT harvesting and ACL reconstruction. Type II was characterised by five layers and its mean distance between the tendons and the patella was 88.81 mm. This distance was shorter than in Type I, but still seems to be sufficient to harvest this tendon. In addition, it is important that the fifth head tendon was independent of the QT and attached very deeply, so it can be safely stated that in this type of tendons, QT harvesting was quite safe. Type III was also characterised by five layers and its mean distance between the tendons and the patella was 78.77 mm. Both the arrangement of its layers and the fairly safe distance make this a safe type for tendon harvesting. On the other hand, Type IV, which was also characterised by five layers, and the mean distance between the tendons and the patella was 134.66 mm. This type seems "ideal" for tendon harvesting as all the accessory tendons of the extra heads fuse with the larger muscles (vasci muscles) and the length of the QT was very long. Finally, Type V was characterised by five layers with a mean distance between the tendons and the patella of 27.92 mm; it also seems to be very complicated and it would be definitely difficult to perform a sufficient cruciate ligament graft harvest of the quadriceps tendon due to its short distance between the tendon junction and the patella.

To determine the correct graft segment length when harvesting a QF tendon, the periosteal sleeve from the patella or a patellar bone block should be deliberated. To improve graft fixation and graft incorporation into the tunnel, ACL reconstruction should include adequate graft length, because poor fixation can result in failed ACL reconstruction. Although bone–patellar tendon–bone autograft currently appears to be the gold standard of ACL reconstruction [26], it is associated with various problems such as patellar fractures [3, 10, 26], patellar tendon ruptures [2, 24], and anterior knee pain [11, 26]]. Hence, QT harvest trials are becoming increasingly popular. Current studies are examining whether it is safer for the patient to harvest the QT with a bone–patellar tendon–bone autograft. So far, no one has taken into account the complexity of the QT structure, which seems very complicated. It is suggested that the structure of the layers or the presence of accessory heads of the QF should be determined first, and only then should the possible site of harvesting for a reconstruction be determined. This should be determined by MRI or ultrasound of the hip or knee region.

There were some limitations to this research. The present study does have some limitations, one being that no sample size calculation was performed; however, the study is the largest cadaver-based study yet performed on the anatomy of the QT. A greater sample size would have been ideal, but the small size (*n* = 128) was attributed to the morphological diversity of the muscle. Nevertheless, the study has a greater sample size and reveals substantial differences relative to prior research [1, 7, 8, 12, 20, 22, 23, 25, 27, 31]. In addition, the sample population was recruited from a particular population of people in the area around Lodz, Poland and Madrid, Spain, where they have spent the better part of their lives. Therefore, more comprehensive studies are required to reveal whether the observed morphological variations are present in larger populations.

## Conclusion

The findings of this study provide a detailed anatomy of the quadriceps tendon including the accessory tendons of the accessory heads of the QT. It turned out that QT may be represented by more layers than previously reported. The accessory heads of the QF contribute to the layering of the QT. The second conclusion of this work is the development of safe distances depending on the types. Not all types are perfect for harvesting.

### Supplementary information

**Additional file 1.**

## References

[1] Andrikoula S, Tokis A, Vasiliadis HS, Georgoulis A (2006). The extensor mechanism of the knee joint: an anatomical study. Knee Surg Sports Traumatol Arthrosc.

[2] Bonamo JJ, Krinick RM, Sporn AA (1984). Rupture of the patellar ligament after use of its central third for anterior cruciate reconstruction. A report of two cases. J Bone Joint Surg Am.

[3] Christen B, Jakob RP (1992). Fractures associated with patellar ligament grafts in cruciate ligament surgery. J Bone Joint Surg Br.

[4] Clinger B, Xerogeanes J, Feller J, Fink C, Runer A, Richter D, Wascher D (2022). Quadriceps tendon autograft for anterior cruciate ligament reconstruction: state of the art. J ISAKOS.

[5] Danaher M, Faucett SC, Endres NK, Geeslin AG (2023). Repair of Quadriceps and Patellar Tendon Tears. Arthroscopy.

[6] Diermeier T, Tisherman R, Hughes J, Tulman M, Baum Coffey E, Fink C (2020). Quadriceps tendon anterior cruciate ligament reconstruction. Knee Surg Sports Traumatol Arthrosc.

[7] Grob K, Ackland T, Kuster MS, Manestar M, Filgueira L (2016). A newly discovered muscle: The tensor of the vastus intermedius. Clin Anat.

[8] Grob K, Manestar M, Filgueira L, Ackland T, Gilbey H, Kuster MS (2016). New insight in the architecture of the quadriceps tendon. J Exp Orthop.

[9] Iriuchishima T, Shirakura K, Yorifuji H, Fu FH (2012). Anatomical evaluation of the rectus femoris tendon and its related structures. Arch Orthop Trauma Surg.

[10] Jarvela T, Kannus P, Jarvinen M (2000). Anterior knee pain 7 years after an anterior cruciate ligament reconstruction with a bone-patellar tendon-bone autograft. Scand J Med Sci Sports.

[11] Kartus J, Magnusson L, Stener S, Brandsson S, Eriksson BI, Karlsson J (1999). Complications following arthroscopic anterior cruciate ligament reconstruction. A 2-5-year follow-up of 604 patients with special emphasis on anterior knee pain. Knee Surg Sports Traumatol Arthrosc.

[12] Krebs N, Yaish A, O’Neill N (2019). Anatomic evaluation of the quadriceps tendon in cadaveric specimens: application for anterior cruciate ligament reconstruction graft choice. Spartan Med Res J.

[13] Meena A, D’Ambrosi R, Runer A, Raj A, Attri M, Abermann E (2023). Quadriceps tendon autograft with or without bone block have comparable clinical outcomes, complications and revision rate for ACL reconstruction: a systematic review. Knee Surg Sports Traumatol Arthrosc.

[14] Olewnik L, Gonera B, Podgorski M, Polguj M, Jezierski H, Topol M (2019). A proposal for a new classification of pes anserinus morphology. Knee Surg Sports Traumatol Arthrosc.

[15] Olewnik L, Karauda P, Gonera B, Kurtys K, Tubbs RS, Paulsen F (2021). Impact of plantaris ligamentous tendon. Sci Rep.

[16] Olewnik Ł, Ruzik K, Szewczyk B, Podgórski M, Aragonés P, Karauda P (2022). The relationship between additional heads of the quadriceps femoris, the vasti muscles, and the patellar ligament. Biomed Res Int.

[17] Olewnik L, Tubbs RS, Ruzik K, Podgorski M, Aragones P, Wasniewska A (2021). Quadriceps or multiceps femoris?-Cadaveric study. Clin Anat.

[18] Olewnik Ł, Zielinska N, Ruzik K, Karauda P, Podgórski M, Borowski A, LaPrade RF (2022). A new look at quadriceps tendon - Is it really composed of three layers?. Knee.

[19] Persson A, Fjeldsgaard K, Gjertsen JE, Kjellsen AB, Engebretsen L, Hole RM, Fevang JM (2014). Increased risk of revision with hamstring tendon grafts compared with patellar tendon grafts after anterior cruciate ligament reconstruction: a study of 12,643 patients from the Norwegian Cruciate Ligament Registry, 2004–2012. Am J Sports Med.

[20] Shea KG, Burlile JF, Richmond CG, Ellis HB, Wilson PL, Fabricant PD (2019). Quadriceps tendon graft anatomy in the skeletally immature patient. Orthop J Sports Med.

[21] Singh H, Glassman I, Sheean A, Hoshino Y, Nagai K, de Sa D (2023). Less than 1% risk of donor-site quadriceps tendon rupture post-ACL reconstruction with quadriceps tendon autograft: a systematic review. Knee Surg Sports Traumatol Arthrosc..

[22] Slone HS, Ashford WB, Xerogeanes JW (2016). Minimally invasive quadriceps tendon harvest and graft preparation for all-inside anterior cruciate ligament reconstruction. Arthrosc Tech.

[23] Slone HS, Romine SE, Premkumar A, Xerogeanes JW (2015). Quadriceps tendon autograft for anterior cruciate ligament reconstruction: a comprehensive review of current literature and systematic review of clinical results. Arthroscopy.

[24] Sonin AH, Fitzgerald SW, Bresler ME, Kirsch MD, Hoff FL, Friedman H (1995). MR imaging appearance of the extensor mechanism of the knee: functional anatomy and injury patterns. Radiographics.

[25] Stäubli H, Jacob RP, Stäubli H (1997). Arthroscopically assisted ACL reconstruction using autologous quadriceps tendon. The Knee and the Cruciate Ligaments.

[26] Strauss M, Kennedy ML, Brady A, Moatshe G, Chahla J, LaPrade RF (2021). Qualitative and quantitative anatomy of the human quadriceps tendon in young cadaveric specimens. Orthop J Sports Med.

[27] Waligora AC, Johanson NA, Hirsch BE (2009). Clinical anatomy of the quadriceps femoris and extensor apparatus of the knee. Clin Orthop Relat Res.

[28] Watson SL, Kingham YE, Patel RM (2022). Chronic quadriceps tendon ruptures: primary repair of quadriceps via bioaugmentation and patellar tendon lengthening. Arthrosc Tech.

[29] Yablon CM, Pai D, Dong Q, Jacobson JA (2014). Magnetic resonance imaging of the extensor mechanism. Magn Reson Imag Clin N Am.

[30] Zakharia A, Lameire DL, Abdel Khalik H, Kay J, Uddandam A, Nagai K (2022). Quadriceps tendon autograft for pediatric anterior cruciate ligament reconstruction results in promising postoperative function and rates of return to sports: A systematic review. Knee Surg Sports Traumatol Arthrosc.

[31] Zeiss J, Saddemi SR, Ebraheim NA (1992). MR imaging of the quadriceps tendon: normal layered configuration and its importance in cases of tendon rupture. AJR Am J Roentgenol.

