# Supplementary material for: The accessory heads of the quadriceps femoris muscle may affect the layering of the quadriceps tendon and potential graft harvest lengths
Source: Knee Surg Sports Traumatol Arthrosc. 2023 Nov 6;31(12):5755–64. doi: 10.1007/s00167-023-07647-x (PMC10719154; doi:10.1007/s00167-023-07647-x)
Supplement: Supplementary file 1 — Additional file 1. [file 167_2023_7647_MOESM1_ESM.docx]

**Supplementary Table. 1.** Morphometric measurements between sexes.

| **PARAMETER** | **SEX** | | |
| --- | --- | --- | --- |
|  | **FEMALE** | **MALE** | **P value** |
| \| Distance between connection of the tendon and patella \| \| --- \| | 84.28 (18.22) | 113.21 (21.27) | 0.0000 |
| \| RF MTJ width \| \| --- \| | 25.46 (9.03) | 27.52 (9.59) | n.s. |
| \| RF MTJ thickness \| \| --- \| | 2.69 (0.30) | 2.85 (0.27) | 0.0042 |
| \|  \| \| --- \|   RF tendon length | 120.64 (50.27) | 137.79 (47.04) | 0.0001 |
| \| RF tendon insertion width \| \| --- \| | 34.35 (9.04) | 37.27 (8.16) | n.s. |
| RF tendon insertion thickness | 2.49 (0.43) | 2.56 (0.37) | n.s. |
| \| VL muscle belly length - superficial part \| \| --- \| | 251.97 (35.18) | 263.92 (35.32) | 0.0375 |
| VL MTJ width - superficial part | 32.77 (11.02) | 37.24 (9.79) | 0.0123 |
| VL MTJ thickness - superficial part | 2.81 (0.31) | 2.86 (0.25) | n.s. |
| \| VL tendon length - superficial part \| \| --- \| | 80.43 (40.17) | 69.32 (34.26) | n.s. |
| VL tendon insertion width - superficial part | 33.03 (9.44) | 30.68 (8.30) | n.s. |
| VL tendon insertion thickness - superficial part | 2.73 (0.34) | 2.77 (0.31) | n.s. |
| VL muscle belly length - intermediate part | 191.64 (47.11) | 206.16 (49.63) | 0.0090 |
| \| VL MTJ width - intermediate part \| \| --- \| | 21.20 (3.98) | 21.64 (3.75) | n.s. |
| \| VL MTJ thickness - intermediate part \| \| --- \| | 2.84 (0.47) | 2.89 (0.52) | n.s. |
| VL tendon length - intermediate part | 132.51 (47.21) | 125.17 (43.81) | n.s. |
| \| VL tendon insertion width - intermediate part \| \| --- \| | 31.26 (8.37) | 31.85 (8.91) | n.s. |
| VL tendon insertion thickness - intermediate part | 2.94 (0.39) | 3.02 (0.32) | n.s. |
| \| VL muscle belly length - deep part \| \| --- \| | 81.63 (12.78) | 84.15 (16.09) | n.s. |
| \| VL MTJ width - deep part \| \| --- \| | 7.51 (2.06) | 8.53 (2.92) | 0.0157 |
| \| VL MTJ thickness - deep part \| \| --- \| | 2.66 (0.50) | 2.69 (0.43) | n.s. |
| VL tendon length - deep part | 59.55 (15.89) | 61.05 (14.21) | n.s. |
| \| VL tendon insertion width - deep part \| \| --- \| | 10.84 (2.18) | 10.97 (1.96) | n.s. |
| \| VL tendon insertion thickness - deep part \| \| --- \| | 2.53 (0.46) | 2.56 (0.44) | n.s. |
| \| VM muscle belly length \| \| --- \| | 276.81 (31.42) | 289.75 (27.35) | 0.0188 |
| \| VM MTJ width \| \| --- \| | 41.53 (7.06) | 41.19 (6.60) | n.s. |
| \| VM MTJ thickness \| \| --- \| | 2.89 (0.31) | 2.95 (0.29) | n.s. |
| \| VM tendon length \| \| --- \| | 35.40 (6.80) | 35.74 (6.63) | n.s. |
| \| VM tendon insertion width \| \| --- \| | 41.65 (5.59) | 42.58 (5.71) | n.s. |
| \| VM tendon insertion thickness \| \| --- \| | 3.13 (0.59) | 3.23 (0.69) | n.s. |
| \| VI muscle belly length \| \| --- \| | 170.98 (64.20) | 163.53 (65.57) | n.s. |
| VI MTJ width | 27.99 (4.46) | 27.49 (5.71) | n.s. |
| VI MTJ thickness | 3.13 (0.52) | 3.15 (0.57) | n.s. |
| \| VI tendon length \|  \| \| --- \| --- \| | 125.63 (64.72) | 137.80 (67.38) | n.s. |
| VI tendon insertion width | 38.54 (8.62) | 37.62 (10.04) | n.s. |
| VI tendon insertion thickness | 3.34 (0.64) | 3.35 (0.75) | n.s. |
| \| 5^th^ head muscle belly length \| \| --- \| | 108.62 (31.28) | 107.56 (29.56) | n.s. |
| 5^th^ head MTJ width | 16.47 (6.34) | 17.12 (6.99) | n.s. |
| 5^th^ MTJ thickness | 2.81 (0.80) | 2.97 (0.97) | n.s. |
| \| 5^th^ tendon length \|  \| \| --- \| --- \| | 196.26 (25.42) | 196.58 (25.31) | n.s. |
| 5^th^ tendon insertion width | 10.65 (3.58) | 10.25 (3.32) | n.s. |
| 5^th^ tendon insertion thickness | 2.03 (0.43) | 2.03 (0.38) | n.s. |

n.s.-non significant

**Supplementary Table 2.** Morphometric measurements between sides.

| **PARAMETER** | **SIDE** | | |
| --- | --- | --- | --- |
|  | **RIGHT** | **LEFT** | **P value** |
| \| Distance between connection of the tendon and patella \| \| --- \| | 98.87 (24.47) | 98.61 (24.72) | n.s. |
| \| RF MTJ width \| \| --- \| | 26.38 (9.42) | 26.60 (9.32) | n.s. |
| \| RF MTJ thickness \| \| --- \| | 2.76 (0.29) | 2.77(0.30) | n.s. |
| \|  \| \| --- \|   RF tendon length | 129.18 (49.47) | 129.25 (49.42) | n.s. |
| \| RF tendon insertion width \| \| --- \| | 35.85 (8.67) | 35.77 (8.80) | n.s. |
| RF tendon insertion thickness | 2.51 (0.41) | 2.53 (0.40) | n.s. |
| \| VL muscle belly length - superficial part \| \| --- \| | 257.88 (35.81) | 258.01 (35.71) | n.s. |
| VL MTJ width - superficial part | 35.12 (10.58) | 34.89 (10.75) | n.s. |
| VL MTJ thickness - superficial part | 2.84 (0.28) | 2.83 (0.29) | n.s. |
| \| VL tendon length - superficial part \| \| --- \| | 74.78 (37.71) | 74.96 (37.78) | n.s. |
| VL tendon insertion width - superficial part | 31.83 (8.93) | 31.88 (9.00) | n.s. |
| VL tendon insertion thickness - superficial part | 2.75 (0.33) | 2.75 (0.33) | n.s. |
| VL muscle belly length - intermediate part | 198.80 (48.95) | 198.99 (48.92) | n.s. |
| \| VL MTJ width - intermediate part \| \| --- \| | 21.30 (3.77) | 21.54 (3.98) | n.s. |
| \| VL MTJ thickness - intermediate part \| \| --- \| | 2.87 (0.47) | 2.86 (0.53) | n.s. |
| VL tendon length - intermediate part | 128.86 (45.69) | 128.82 (45.71) | n.s. |
| \| VL tendon insertion width - intermediate part \| \| --- \| | 31.53 (8.65) | 31.58 (8.65) | n.s. |
| VL tendon insertion thickness - intermediate part | 2.97 (0.35) | 2.99 (0.37) | n.s. |
| \| VL muscle belly length - deep part \| \| --- \| | 82.89(14.57) | 82.89 (14.60) | n.s. |
| \| VL MTJ width - deep part \| \| --- \| | 8.0205 (2.56743) | 8.02 (2.60) | n.s. |
| \| VL MTJ thickness - deep part \| \| --- \| | 2.68 (0.46) | 2.68 (0.48) | n.s. |
| VL tendon length - deep part | 60.29 (15.11) | 60.32 (15.08) | n.s. |
| \| VL tendon insertion width - deep part \| \| --- \| | 10.91 (2.08) | 10.90 (2.06) | n.s. |
| \| VL tendon insertion thickness - deep part \| \| --- \| | 2.54 (0.44) | 2.54 (0.46) | n.s. |
| \| VM muscle belly length \| \| --- \| | 283.25 (30.18) | 283.31 (30.15) | n.s. |
| \| VM MTJ width \| \| --- \| | 41.40 (6.77) | 41.31 (6.90) | n.s. |
| \| VM MTJ thickness \| \| --- \| | 2.90 (0.30) | 2.93 (0.29) | n.s. |
| \| VM tendon length \| \| --- \| | 35.54 (6.75) | 35.61 (6.68) | n.s. |
| \| VM tendon insertion width \| \| --- \| | 42.09 (5.65) | 42.14 (5.69) | n.s. |
| \| VM tendon insertion thickness \| \| --- \| | 3.17 (0.63) | 3.20 (0.66) | n.s. |
| \| VI muscle belly length \| \| --- \| | 167.23 (64.98) | 167.28 (65.013) | n.s. |
| VI MTJ width | 27.80 (5.20) | 27.68 (5.06) | n.s. |
| VI MTJ thickness | 3.14 (0.54) | 3.15 (0.55) | n.s. |
| \| VI tendon length \|  \| \| --- \| --- \| | 131.65 (66.36) | 131.78 (66.32) | n.s. |
| VI tendon insertion width | 38.06 (9.36) | 38.10 (9.37) | n.s. |
| VI tendon insertion thickness | 3.34 (0.68) | 3.35 (0.71) | n.s. |
| \| 5^th^ head muscle belly length \| \| --- \| | 109.06 (29.96) | 107.10 (30.77) | n.s. |
| 5^th^ head MTJ width | 16.88 (6.77) | 16.74 (6.63) | n.s. |
| 5^th^ MTJ thickness | 2.91 (0.93) | 2.88 (0.86) | n.s. |
| \| 5^th^ tendon length \|  \| \| --- \| --- \| | 196.58 (25.57) | 196.29 (25.16) | n.s. |
| 5^th^ tendon insertion width | 10.42 (3.43) | 10.45 (3.47) | n.s. |
| 5^th^ tendon insertion thickness | 2.01 (0.40) | 2.04 (0.41) | n.s. |

**Supplementary Table. 3.** The relationships between the types

| **PARAMETER** | **TYPE** | | | | | |
| --- | --- | --- | --- | --- | --- | --- |
|  | **1** | **2** | **3** | **4** | **5** | **P value** |
| \| Distance between connection of the tendon and patella \| \| --- \| | 109.55 (17.56) | 88.81 (11.74) | 78.77 (23.44) | 134.46 (9.54) | 27.92 (3.16) | 0.0001 |
| \| RF MTJ width \| \| --- \| | 20.91 (5.26) | 37.24 (6.87) | 28.12 (9.42) | 23.05 (0.25) | 29.17 (1.31) | 0.0001 |
| \| RF MTJ thickness \| \| --- \| | 2.83 (0.30) | 2.70 (0.18) | 2.83 (0.26) | 2.01 (0.03) | 2.81 (0.09) | 0.0001 |
| \|  \| \| --- \|   RF tendon length | 94.56 (17.10) | 186.45 (17.25) | 123.07 (55.46) | 195.53 (1.04) | 198.80 (3.90) | 0.0001 |
| \| RF tendon insertion width \| \| --- \| | 29.79 (6.22) | 45.57 (1.57) | 39.29 (4.28) | 46.82 (0.08) | 34.01 (1.96) | 0.0001 |
| RF tendon insertion thickness | 2.66 (0.33) | 2.23 (0.32) | 2.51 (0.57) | 2.61 (0.10) | 2.53 (0.54) | 0.0001 |
| \| VL muscle belly length - superficial part \| \| --- \| | 245.17 (39.69) | 278.19 (2.30) | 246.64 (22.71) | 308.97 (0.61) | 296.16 (0.56) | 0.0001 |
| VL MTJ width - superficial part | 33.14 (10.57) | 40.83 (10.14) | 33.41 (9.12) | 33.42 (0.03) | 24.33 (0.29) | 0.0056 |
| VL MTJ thickness - superficial part | 2.80 (0.28) | 2.84 (0.25) | 3.03 (0.35) | 2.85 (0.02) | 2.69 (0.23) | n.s. |
| \| VL tendon length - superficial part \| \| --- \| | 90.78 (35.36) | 42.91 (7.60) | 92.91 (42.14) | 42.18 (0.20) | 41.69 (1.90) | 0.0001 |
| VL tendon insertion width - superficial part | 35.09 (5.62) | 24.89 (9.85) | 33.19 (10.88) | 39.99 (0.03) | 22.65 (1.86) | 0.0001 |
| VL tendon insertion thickness - superficial part | 2.72 (0.27) | 2.81 (0.40) | 2.83 (0.38) | 2.77 (0.03) | 2.47 (0.43) | n.s. |
| VL muscle belly length - intermediate part | 208.55 (37.48) | 185.03 (58.92) | 171.13 (33.32) | 149.79 (0.69) | 295.24 (1.32) | 0.0001 |
| \| VL MTJ width - intermediate part \| \| --- \| | 20.99 (4.43) | 23.03 (1.62) | 21.85 (1.06) | 22.14 (0.08) | 12.82 (1.97) | 0.0004 |
| \| VL MTJ thickness - intermediate part \| \| --- \| | 2.67 (0.49) | 3.19 (0.36) | 3.06 (0.33) | 3.07 (0.03) | 2.57 (0.61) | 0.0001 |
| VL tendon length - intermediate part | 133.76 (26.26) | 122.04 (68.75) | 141.86 (45.48) | 128.88 (0.19) | 55.48 (1.21) | 0.0001 |
| \| VL tendon insertion width - intermediate part \| \| --- \| | 36.38 (5.93) | 23.87 (1.94) | 34.71 (9.08) | 21.32 (0.17) | 12.32 (1.34) | 0.0001 |
| VL tendon insertion thickness - intermediate part | 2.85 (0.24) | 3.30 (0.34) | 2.90 (0.23) | 3.37 (0.03) | 2.43 (0.37) | 0.0001 |
| \| VL muscle belly length - deep part \| \| --- \| | 86.77 (18.48) | 78.34 (2.78) | 75.41 (1.69) | 82.36 (0.13) | 80.54 (2.08) | 0.0001 |
| \| VL MTJ width - deep part \| \| --- \| | 8.64 (3.24) | 7.55 (0.54) | 7.39 (0.34) | 6.42 (0.25) | 4.85 (0.27) | 0.0041 |
| \| VL MTJ thickness - deep part \| \| --- \| | 2.44 (0.47) | 3.02 (0.22) | 2.99 (0.28) | 2.87 (0.05) | 2.72 (0.05) | 0.0001 |
| VL tendon length - deep part | 51.74 (11.92) | 76.27 (2.56) | 55.40 (13.36) | 75.21 (0.16) | 74.81 (3.07) | 0.0001 |
| \| VL tendon insertion width - deep part \| \| --- \| | 10.93 (2.51) | 11.49 (0.83) | 10.18 (1.23) | 10.27 (0.07) | 8.37 (1.89) | 0.0189 |
| \| VL tendon insertion thickness - deep part \| \| --- \| | 2.41 (0.47) | 2.75 (0.24) | 2.94 (0.24) | 2.42 (0.12) | 1.88 (0.34) | 0.0001 |
| \| VM muscle belly length \| \| --- \| | 268.35 (30.72) | 306.52 (12.96) | 290.46 (15.43) | 315.99 (0.08) | 287.14 (2.62) | 0.0001 |
| \| VM MTJ width \| \| --- \| | 40.17 (7.39) | 43.14 (5.41) | 40.96 (1.54) | 54.34 (0.14) | 35.14 (1.59) | 0.0023 |
| \| VM MTJ thickness \| \| --- \| | 2.80 (0.27) | 3.08 (0.24) | 3.00 (0.37) | 3.23 (0.07) | 3.02 (0.20) | 0.0001 |
| \| VM tendon length \| \| --- \| | 38.11 (6.73) | 30.62 (2.37) | 36.75 (5.46) | 40.64 (0.15) | 24.65 (2.36) | 0.0001 |
| \| VM tendon insertion width \| \| --- \| | 42.59 (6.48) | 40.30 (1.64) | 42.72 (1.66) | 54.66 (0.68) | 34.88 (2.00) | 0.0001 |
| \| VM tendon insertion thickness \| \| --- \| | 2.75 (0.30) | 3.88 (0.47) | 3.74 (0.49) | 3.13 (0.04) | 2.75 (0.19) | 0.0001 |
| \| VI muscle belly length \| \| --- \| | 206.66 (41.0) | 84.44 (18.29) | 163.79 (56.51) | 154.01 (0.87) | 217.70 (2.20) | 0.0001 |
| VI MTJ width | 25.44 (3.77) | 29.62 (5.34) | 30.34 (4.22) | 37.58 (0.44) | 33.17 (2.91) | 0.0001 |
| VI MTJ thickness | 2.80 (0.25) | 3.80 (0.33) | 3.32 (0.57) | 3.38 (0.04) | 2.73 (0.30) | 0.0001 |
| \| VI tendon length \|  \| \| --- \| --- \| | 99.70 (57.42) | 205.30 (22.17) | 120.47 (54.20) | 130.12 (0.21) | 97.04 (0.63) | 0.0001 |
| VI tendon insertion width | 33.99 (8.56) | 46.83 (2.14) | 39.47 (7.11) | 45.11 (0.37) | 22.21 (1.29) | 0.0001 |
| VI tendon insertion thickness | 2.92 (0.56) | 3.98 (0.41) | 3.87 (0.32) | 3.87 (0.05) | 3.03 (0.16) | 0.0001 |
| \| 5^th^ head muscle belly length \| \| --- \| |  | 108.70 (20.73) | 82.53 (9.27) | 188.51 (0.63) | 111.42 (1.33) | 0.0001 |
| 5^th^ head MTJ width |  | 12.94 (0.75) | 28.18 (0.65) | 11.69 (0.04) | 16.06 (0.45) | 0.0001 |
| 5^th^ MTJ thickness |  | 2.46 (0.32) | 4.34 (0.28) | 2.59 (0.03) | 1.95 (0.10) | 0.0001 |
| \| 5^th^ tendon length \|  \| \| --- \| --- \| |  | 191.24 (23.26) | 191.22 (3.52) | 198.01 (0.24) | 258.55 (4.32) | 0.0002 |
| 5^th^ tendon insertion width |  | 12.60 (0.85) | 4.85 (0.33) | 12.26 (0.12) | 9.19 (1.71) | 0.0001 |
| 5^th^ tendon insertion thickness |  | 2.14 (0.16) | 1.53 (0.10) | 2.97 (0.05) | 1.85 (0.31) | 0.0001 |

**Table. 1.** Morphometric measurements between sexes.

| **PARAMETER** | **SEX** | | |
| --- | --- | --- | --- |
|  | **FEMALE** | **MALE** | **P value** |
| \| Distance between connection of the tendon and patella \| \| --- \| | 84.28 (18.22) | 113.21 (21.27) | 0.0000 |
| \| RF MTJ width \| \| --- \| | 25.46 (9.03) | 27.52 (9.59) | n.s. |
| \| RF MTJ thickness \| \| --- \| | 2.69 (0.30) | 2.85 (0.27) | 0.0042 |
| \|  \| \| --- \|   RF tendon length | 120.64 (50.27) | 137.79 (47.04) | 0.0001 |
| \| RF tendon insertion width \| \| --- \| | 34.35 (9.04) | 37.27 (8.16) | n.s. |
| RF tendon insertion thickness | 2.49 (0.43) | 2.56 (0.37) | n.s. |
| \| VL muscle belly length - superficial part \| \| --- \| | 251.97 (35.18) | 263.92 (35.32) | 0.0375 |
| VL MTJ width - superficial part | 32.77 (11.02) | 37.24 (9.79) | 0.0123 |
| VL MTJ thickness - superficial part | 2.81 (0.31) | 2.86 (0.25) | n.s. |
| \| VL tendon length - superficial part \| \| --- \| | 80.43 (40.17) | 69.32 (34.26) | n.s. |
| VL tendon insertion width - superficial part | 33.03 (9.44) | 30.68 (8.30) | n.s. |
| VL tendon insertion thickness - superficial part | 2.73 (0.34) | 2.77 (0.31) | n.s. |
| VL muscle belly length - intermediate part | 191.64 (47.11) | 206.16 (49.63) | 0.0090 |
| \| VL MTJ width - intermediate part \| \| --- \| | 21.20 (3.98) | 21.64 (3.75) | n.s. |
| \| VL MTJ thickness - intermediate part \| \| --- \| | 2.84 (0.47) | 2.89 (0.52) | n.s. |
| VL tendon length - intermediate part | 132.51 (47.21) | 125.17 (43.81) | n.s. |
| \| VL tendon insertion width - intermediate part \| \| --- \| | 31.26 (8.37) | 31.85 (8.91) | n.s. |
| VL tendon insertion thickness - intermediate part | 2.94 (0.39) | 3.02 (0.32) | n.s. |
| \| VL muscle belly length - deep part \| \| --- \| | 81.63 (12.78) | 84.15 (16.09) | n.s. |
| \| VL MTJ width - deep part \| \| --- \| | 7.51 (2.06) | 8.53 (2.92) | 0.0157 |
| \| VL MTJ thickness - deep part \| \| --- \| | 2.66 (0.50) | 2.69 (0.43) | n.s. |
| VL tendon length - deep part | 59.55 (15.89) | 61.05 (14.21) | n.s. |
| \| VL tendon insertion width - deep part \| \| --- \| | 10.84 (2.18) | 10.97 (1.96) | n.s. |
| \| VL tendon insertion thickness - deep part \| \| --- \| | 2.53 (0.46) | 2.56 (0.44) | n.s. |
| \| VM muscle belly length \| \| --- \| | 276.81 (31.42) | 289.75 (27.35) | 0.0188 |
| \| VM MTJ width \| \| --- \| | 41.53 (7.06) | 41.19 (6.60) | n.s. |
| \| VM MTJ thickness \| \| --- \| | 2.89 (0.31) | 2.95 (0.29) | n.s. |
| \| VM tendon length \| \| --- \| | 35.40 (6.80) | 35.74 (6.63) | n.s. |
| \| VM tendon insertion width \| \| --- \| | 41.65 (5.59) | 42.58 (5.71) | n.s. |
| \| VM tendon insertion thickness \| \| --- \| | 3.13 (0.59) | 3.23 (0.69) | n.s. |
| \| VI muscle belly length \| \| --- \| | 170.98 (64.20) | 163.53 (65.57) | n.s. |
| VI MTJ width | 27.99 (4.46) | 27.49 (5.71) | n.s. |
| VI MTJ thickness | 3.13 (0.52) | 3.15 (0.57) | n.s. |
| \| VI tendon length \|  \| \| --- \| --- \| | 125.63 (64.72) | 137.80 (67.38) | n.s. |
| VI tendon insertion width | 38.54 (8.62) | 37.62 (10.04) | n.s. |
| VI tendon insertion thickness | 3.34 (0.64) | 3.35 (0.75) | n.s. |
| \| 5^th^ head muscle belly length \| \| --- \| | 108.62 (31.28) | 107.56 (29.56) | n.s. |
| 5^th^ head MTJ width | 16.47 (6.34) | 17.12 (6.99) | n.s. |
| 5^th^ MTJ thickness | 2.81 (0.80) | 2.97 (0.97) | n.s. |
| \| 5^th^ tendon length \|  \| \| --- \| --- \| | 196.26 (25.42) | 196.58 (25.31) | n.s. |
| 5^th^ tendon insertion width | 10.65 (3.58) | 10.25 (3.32) | n.s. |
| 5^th^ tendon insertion thickness | 2.03 (0.43) | 2.03 (0.38) | n.s. |

n.s.-non significant

**Table 2.** Morphometric measurements between sides.

| **PARAMETER** | **SIDE** | | |
| --- | --- | --- | --- |
|  | **RIGHT** | **LEFT** | **P value** |
| \| Distance between connection of the tendon and patella \| \| --- \| | 98.87 (24.47) | 98.61 (24.72) | n.s. |
| \| RF MTJ width \| \| --- \| | 26.38 (9.42) | 26.60 (9.32) | n.s. |
| \| RF MTJ thickness \| \| --- \| | 2.76 (0.29) | 2.77(0.30) | n.s. |
| \|  \| \| --- \|   RF tendon length | 129.18 (49.47) | 129.25 (49.42) | n.s. |
| \| RF tendon insertion width \| \| --- \| | 35.85 (8.67) | 35.77 (8.80) | n.s. |
| RF tendon insertion thickness | 2.51 (0.41) | 2.53 (0.40) | n.s. |
| \| VL muscle belly length - superficial part \| \| --- \| | 257.88 (35.81) | 258.01 (35.71) | n.s. |
| VL MTJ width - superficial part | 35.12 (10.58) | 34.89 (10.75) | n.s. |
| VL MTJ thickness - superficial part | 2.84 (0.28) | 2.83 (0.29) | n.s. |
| \| VL tendon length - superficial part \| \| --- \| | 74.78 (37.71) | 74.96 (37.78) | n.s. |
| VL tendon insertion width - superficial part | 31.83 (8.93) | 31.88 (9.00) | n.s. |
| VL tendon insertion thickness - superficial part | 2.75 (0.33) | 2.75 (0.33) | n.s. |
| VL muscle belly length - intermediate part | 198.80 (48.95) | 198.99 (48.92) | n.s. |
| \| VL MTJ width - intermediate part \| \| --- \| | 21.30 (3.77) | 21.54 (3.98) | n.s. |
| \| VL MTJ thickness - intermediate part \| \| --- \| | 2.87 (0.47) | 2.86 (0.53) | n.s. |
| VL tendon length - intermediate part | 128.86 (45.69) | 128.82 (45.71) | n.s. |
| \| VL tendon insertion width - intermediate part \| \| --- \| | 31.53 (8.65) | 31.58 (8.65) | n.s. |
| VL tendon insertion thickness - intermediate part | 2.97 (0.35) | 2.99 (0.37) | n.s. |
| \| VL muscle belly length - deep part \| \| --- \| | 82.89(14.57) | 82.89 (14.60) | n.s. |
| \| VL MTJ width - deep part \| \| --- \| | 8.0205 (2.56743) | 8.02 (2.60) | n.s. |
| \| VL MTJ thickness - deep part \| \| --- \| | 2.68 (0.46) | 2.68 (0.48) | n.s. |
| VL tendon length - deep part | 60.29 (15.11) | 60.32 (15.08) | n.s. |
| \| VL tendon insertion width - deep part \| \| --- \| | 10.91 (2.08) | 10.90 (2.06) | n.s. |
| \| VL tendon insertion thickness - deep part \| \| --- \| | 2.54 (0.44) | 2.54 (0.46) | n.s. |
| \| VM muscle belly length \| \| --- \| | 283.25 (30.18) | 283.31 (30.15) | n.s. |
| \| VM MTJ width \| \| --- \| | 41.40 (6.77) | 41.31 (6.90) | n.s. |
| \| VM MTJ thickness \| \| --- \| | 2.90 (0.30) | 2.93 (0.29) | n.s. |
| \| VM tendon length \| \| --- \| | 35.54 (6.75) | 35.61 (6.68) | n.s. |
| \| VM tendon insertion width \| \| --- \| | 42.09 (5.65) | 42.14 (5.69) | n.s. |
| \| VM tendon insertion thickness \| \| --- \| | 3.17 (0.63) | 3.20 (0.66) | n.s. |
| \| VI muscle belly length \| \| --- \| | 167.23 (64.98) | 167.28 (65.013) | n.s. |
| VI MTJ width | 27.80 (5.20) | 27.68 (5.06) | n.s. |
| VI MTJ thickness | 3.14 (0.54) | 3.15 (0.55) | n.s. |
| \| VI tendon length \|  \| \| --- \| --- \| | 131.65 (66.36) | 131.78 (66.32) | n.s. |
| VI tendon insertion width | 38.06 (9.36) | 38.10 (9.37) | n.s. |
| VI tendon insertion thickness | 3.34 (0.68) | 3.35 (0.71) | n.s. |
| \| 5^th^ head muscle belly length \| \| --- \| | 109.06 (29.96) | 107.10 (30.77) | n.s. |
| 5^th^ head MTJ width | 16.88 (6.77) | 16.74 (6.63) | n.s. |
| 5^th^ MTJ thickness | 2.91 (0.93) | 2.88 (0.86) | n.s. |
| \| 5^th^ tendon length \|  \| \| --- \| --- \| | 196.58 (25.57) | 196.29 (25.16) | n.s. |
| 5^th^ tendon insertion width | 10.42 (3.43) | 10.45 (3.47) | n.s. |
| 5^th^ tendon insertion thickness | 2.01 (0.40) | 2.04 (0.41) | n.s. |

**Table. 3.** The relationships between the types

| **PARAMETER** | **TYPE** | | | | | |
| --- | --- | --- | --- | --- | --- | --- |
|  | **1** | **2** | **3** | **4** | **5** | **P value** |
| \| Distance between connection of the tendon and patella \| \| --- \| | 109.55 (17.56) | 88.81 (11.74) | 78.77 (23.44) | 134.46 (9.54) | 27.92 (3.16) | 0.0001 |
| \| RF MTJ width \| \| --- \| | 20.91 (5.26) | 37.24 (6.87) | 28.12 (9.42) | 23.05 (0.25) | 29.17 (1.31) | 0.0001 |
| \| RF MTJ thickness \| \| --- \| | 2.83 (0.30) | 2.70 (0.18) | 2.83 (0.26) | 2.01 (0.03) | 2.81 (0.09) | 0.0001 |
| \|  \| \| --- \|   RF tendon length | 94.56 (17.10) | 186.45 (17.25) | 123.07 (55.46) | 195.53 (1.04) | 198.80 (3.90) | 0.0001 |
| \| RF tendon insertion width \| \| --- \| | 29.79 (6.22) | 45.57 (1.57) | 39.29 (4.28) | 46.82 (0.08) | 34.01 (1.96) | 0.0001 |
| RF tendon insertion thickness | 2.66 (0.33) | 2.23 (0.32) | 2.51 (0.57) | 2.61 (0.10) | 2.53 (0.54) | 0.0001 |
| \| VL muscle belly length - superficial part \| \| --- \| | 245.17 (39.69) | 278.19 (2.30) | 246.64 (22.71) | 308.97 (0.61) | 296.16 (0.56) | 0.0001 |
| VL MTJ width - superficial part | 33.14 (10.57) | 40.83 (10.14) | 33.41 (9.12) | 33.42 (0.03) | 24.33 (0.29) | 0.0056 |
| VL MTJ thickness - superficial part | 2.80 (0.28) | 2.84 (0.25) | 3.03 (0.35) | 2.85 (0.02) | 2.69 (0.23) | n.s. |
| \| VL tendon length - superficial part \| \| --- \| | 90.78 (35.36) | 42.91 (7.60) | 92.91 (42.14) | 42.18 (0.20) | 41.69 (1.90) | 0.0001 |
| VL tendon insertion width - superficial part | 35.09 (5.62) | 24.89 (9.85) | 33.19 (10.88) | 39.99 (0.03) | 22.65 (1.86) | 0.0001 |
| VL tendon insertion thickness - superficial part | 2.72 (0.27) | 2.81 (0.40) | 2.83 (0.38) | 2.77 (0.03) | 2.47 (0.43) | n.s. |
| VL muscle belly length - intermediate part | 208.55 (37.48) | 185.03 (58.92) | 171.13 (33.32) | 149.79 (0.69) | 295.24 (1.32) | 0.0001 |
| \| VL MTJ width - intermediate part \| \| --- \| | 20.99 (4.43) | 23.03 (1.62) | 21.85 (1.06) | 22.14 (0.08) | 12.82 (1.97) | 0.0004 |
| \| VL MTJ thickness - intermediate part \| \| --- \| | 2.67 (0.49) | 3.19 (0.36) | 3.06 (0.33) | 3.07 (0.03) | 2.57 (0.61) | 0.0001 |
| VL tendon length - intermediate part | 133.76 (26.26) | 122.04 (68.75) | 141.86 (45.48) | 128.88 (0.19) | 55.48 (1.21) | 0.0001 |
| \| VL tendon insertion width - intermediate part \| \| --- \| | 36.38 (5.93) | 23.87 (1.94) | 34.71 (9.08) | 21.32 (0.17) | 12.32 (1.34) | 0.0001 |
| VL tendon insertion thickness - intermediate part | 2.85 (0.24) | 3.30 (0.34) | 2.90 (0.23) | 3.37 (0.03) | 2.43 (0.37) | 0.0001 |
| \| VL muscle belly length - deep part \| \| --- \| | 86.77 (18.48) | 78.34 (2.78) | 75.41 (1.69) | 82.36 (0.13) | 80.54 (2.08) | 0.0001 |
| \| VL MTJ width - deep part \| \| --- \| | 8.64 (3.24) | 7.55 (0.54) | 7.39 (0.34) | 6.42 (0.25) | 4.85 (0.27) | 0.0041 |
| \| VL MTJ thickness - deep part \| \| --- \| | 2.44 (0.47) | 3.02 (0.22) | 2.99 (0.28) | 2.87 (0.05) | 2.72 (0.05) | 0.0001 |
| VL tendon length - deep part | 51.74 (11.92) | 76.27 (2.56) | 55.40 (13.36) | 75.21 (0.16) | 74.81 (3.07) | 0.0001 |
| \| VL tendon insertion width - deep part \| \| --- \| | 10.93 (2.51) | 11.49 (0.83) | 10.18 (1.23) | 10.27 (0.07) | 8.37 (1.89) | 0.0189 |
| \| VL tendon insertion thickness - deep part \| \| --- \| | 2.41 (0.47) | 2.75 (0.24) | 2.94 (0.24) | 2.42 (0.12) | 1.88 (0.34) | 0.0001 |
| \| VM muscle belly length \| \| --- \| | 268.35 (30.72) | 306.52 (12.96) | 290.46 (15.43) | 315.99 (0.08) | 287.14 (2.62) | 0.0001 |
| \| VM MTJ width \| \| --- \| | 40.17 (7.39) | 43.14 (5.41) | 40.96 (1.54) | 54.34 (0.14) | 35.14 (1.59) | 0.0023 |
| \| VM MTJ thickness \| \| --- \| | 2.80 (0.27) | 3.08 (0.24) | 3.00 (0.37) | 3.23 (0.07) | 3.02 (0.20) | 0.0001 |
| \| VM tendon length \| \| --- \| | 38.11 (6.73) | 30.62 (2.37) | 36.75 (5.46) | 40.64 (0.15) | 24.65 (2.36) | 0.0001 |
| \| VM tendon insertion width \| \| --- \| | 42.59 (6.48) | 40.30 (1.64) | 42.72 (1.66) | 54.66 (0.68) | 34.88 (2.00) | 0.0001 |
| \| VM tendon insertion thickness \| \| --- \| | 2.75 (0.30) | 3.88 (0.47) | 3.74 (0.49) | 3.13 (0.04) | 2.75 (0.19) | 0.0001 |
| \| VI muscle belly length \| \| --- \| | 206.66 (41.0) | 84.44 (18.29) | 163.79 (56.51) | 154.01 (0.87) | 217.70 (2.20) | 0.0001 |
| VI MTJ width | 25.44 (3.77) | 29.62 (5.34) | 30.34 (4.22) | 37.58 (0.44) | 33.17 (2.91) | 0.0001 |
| VI MTJ thickness | 2.80 (0.25) | 3.80 (0.33) | 3.32 (0.57) | 3.38 (0.04) | 2.73 (0.30) | 0.0001 |
| \| VI tendon length \|  \| \| --- \| --- \| | 99.70 (57.42) | 205.30 (22.17) | 120.47 (54.20) | 130.12 (0.21) | 97.04 (0.63) | 0.0001 |
| VI tendon insertion width | 33.99 (8.56) | 46.83 (2.14) | 39.47 (7.11) | 45.11 (0.37) | 22.21 (1.29) | 0.0001 |
| VI tendon insertion thickness | 2.92 (0.56) | 3.98 (0.41) | 3.87 (0.32) | 3.87 (0.05) | 3.03 (0.16) | 0.0001 |
| \| 5^th^ head muscle belly length \| \| --- \| |  | 108.70 (20.73) | 82.53 (9.27) | 188.51 (0.63) | 111.42 (1.33) | 0.0001 |
| 5^th^ head MTJ width |  | 12.94 (0.75) | 28.18 (0.65) | 11.69 (0.04) | 16.06 (0.45) | 0.0001 |
| 5^th^ MTJ thickness |  | 2.46 (0.32) | 4.34 (0.28) | 2.59 (0.03) | 1.95 (0.10) | 0.0001 |
| \| 5^th^ tendon length \|  \| \| --- \| --- \| |  | 191.24 (23.26) | 191.22 (3.52) | 198.01 (0.24) | 258.55 (4.32) | 0.0002 |
| 5^th^ tendon insertion width |  | 12.60 (0.85) | 4.85 (0.33) | 12.26 (0.12) | 9.19 (1.71) | 0.0001 |
| 5^th^ tendon insertion thickness |  | 2.14 (0.16) | 1.53 (0.10) | 2.97 (0.05) | 1.85 (0.31) | 0.0001 |
